# Essentiality of dietary cholesterol and its interactions with phospholipid in juvenile slipper lobster (*Thenus australiensis*)

**DOI:** 10.1038/s41598-024-60367-1

**Published:** 2024-05-06

**Authors:** Michael J. Landman, Basseer M. Codabaccus, David S. Nichols, Chris G. Carter, Quinn P. Fitzgibbon, Gregory G. Smith

**Affiliations:** 1 Institute for Marine and Antarctic Studies, University of Tasmania, Private Bag 49, Hobart, TAS 7001 Australia; 2 Central Science Laboratory, University of Tasmania, Hobart, TAS Australia

**Keywords:** Slipper lobster, Cholesterol, Phospholipid, Nutrition, Growth, Marine biology, Biochemistry, Developmental biology, Feeding behaviour

## Abstract

This study was conducted to verify the essentiality of dietary cholesterol for early juvenile slipper lobster, *Thenus australiensis* (initial weight 4.50 ± 0.72 g, mean ± SD, CV = 0.16), and to explore the potential for interactions between dietary cholesterol and phospholipid. An 8-week experiment was conducted using six experimental feeds containing three supplemental cholesterol concentrations (0, 0.2 and 0.4% dry matter) at two supplemental phospholipid concentrations (0% and 1.0% dry matter). Dietary cholesterol concentrations of ≥ 0.2% resulted in up to threefold greater weight gain compared to 0% dietary cholesterol, but without any significant main or interactive dietary phospholipid effect. An interaction was observed for lobster survival with lowest survival (46%) recorded for combined 0% cholesterol and 0% phospholipid compared to every other treatment (71–100%). However, all surviving lobsters at 0% dietary cholesterol, regardless of dietary phospholipid level, were in poor nutritional condition. Apparent feed intake (AFI) was significantly higher at dietary cholesterol ≥ 0.2% but was lower for each corresponding dietary cholesterol level at 1% dietary phospholipid. This implied that the feed conversion ratio was improved with supplemental phospholipid. In conclusion, this study confirms the essential nature of dietary cholesterol and that dietary phospholipid can provide additional benefits.

## Introduction

Crustaceans appear to have limited capacity for de novo synthesis of cholesterol (Chol) from low molecular weight precursors such acetate or mevalonate and so must obtain it from the diet^1–3^. As the prevailing sterol in animals, Chol is involved in lipid absorption and transport, is the principle non-polar lipid component in cell membranes, performs a variety of essential roles in cellular signalling processes and, perhaps most notably for crustaceans, is a precursor for steroid hormones involved in moulting and reproduction^3,4^. There is a solid body of research examining dietary Chol requirements and effects in various decapod crustacean groups, in particular for penaeid shrimp^5,6^, palaemonid shrimp^7–10^, crabs^11–15^, crayfish^16,17^, homarid lobsters^18–21^ and palinurid lobsters^22^. While Chol is generally considered essential for normal growth, development and survival, dietary requirements have been shown to vary considerably between species, life stages, and through nutrient interactions.

Dietary Chol may have less importance in the later life stages of some decapod species, as found for subadult Pacific white shrimp, *Litopenaeus vannamei*^6^ and adult American lobster, *Homarus americanus*^19^. For larval and juvenile animals, dietary Chol requirements between 0.1 and 2.1% of feed dry matter (DM) have been reported^3^. However, numerous studies show optimal Chol requirements for a broad range of decapods at generally low inclusion amounts of between 0.1 and 0.5% DM in the diet^8,13,17,18,22,23^. Furthermore, higher dietary Chol inclusion of between 0.8 and 2.0% DM in the diet can have negative effects on growth and survival in some species, such as post-larval Pacific white shrimp^24^, juvenile banana shrimp, *Penaeus merguiensis*^25^ juvenile redclaw crayfish, *Cherax quadricarinatus*^17^, and the megalopa larvae and juveniles of the mud crab, *Scylla serrata*^11,13,14^.

D’Abramo^9^ suggests that omnivorous species may be less dependent on dietary Chol than carnivorous species through an ability to more efficiently utilise phytosterols. For example, requirements for American lobster, an opportunistic carnivore, may be more specific to Chol^21^, while freshwater prawn, *Marcrobrachium rosenbergii*, and Pacific white shrimp have been shown capable of effectively utilising some phytosterols^6,9,26^. Indeed, many decapods appear to have at least some ability to convert other sterols to Chol, through a process of dealkylation, though Chol may still be nutritionally superior for some species and particularly during early development^2^. Likewise, dietary phospholipid and Chol interactions may be particularly important in decapods since phospholipids are often also described as essential nutrients^27^, though the experimental evidence for this is not particularly strong in scyllarid lobsters^28^. It has been proposed that dietary phospholipids may provide additional nutritional benefits through Chol ‘sparing’ effects, the extent to which may be dependent on the species, phospholipid type and combination, and the dietary levels examined^3^.

It is important to establish nutrient essentiality and quantitative requirements on a case-by-case basis, particularly for high-value aquaculture species where rapid growth, cost-effective feeds and waste minimisation are key to economic and environmental sustainability^29^. Cholesterol is known to be a particularly expensive feed ingredient and, even when included at relatively low concentrations, may represent a significant proportion of total feed costs^3^. Despite the cost and apparent importance of dietary Chol in decapods, requirements have not been investigated specifically for *Thenus australiensis* or other scyllarid lobsters and there is limited data available for closely related groups such as the palinurid lobsters^22^.

The primary objective of this study was to test the hypothesis that dietary Chol is essential for normal growth and development in early juvenile *T. australiensis*, and further to explore the potential for interactions between dietary Chol and phospholipid (PhosL). This was examined under standard culture conditions over 8 consecutive weeks using six semi-purified experimental feeds in a 3 × 2 factorial design using three supplemental dietary Chol levels at two different supplemental PhosL levels. To our knowledge, this is the first study to examine dietary Chol effects and its interaction with PhosL in the slipper lobster, *T. australiensis*.

## Results

**Figure 1:**
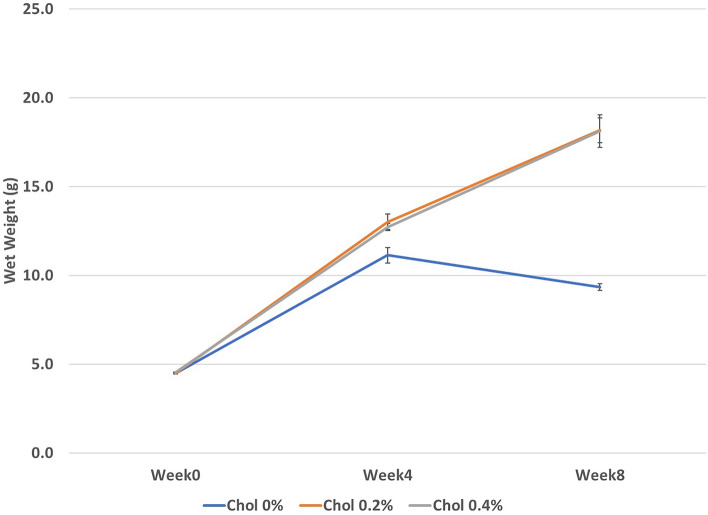
Mean (± SEM, n = 6) wet weight (g) of juvenile *T. australiensis* at three cholesterol (Chol, %) supplemental dietary concentrations after 0, 4 and 8 weeks. Mean weights were calculated using individual lobster weights at weeks 0 and 8. Mean weights at week 4 were estimated from bulk weighing.

A significant dietary Chol effect was found for all general growth endpoints including daily weight gain (WG), total body weight gain (BWG), specific growth rate (SGR) and hepatosomatic index (HPSI) and moulting (Table 1). Dietary Chol of ≥ 0.2% dry matter (DM) resulted in significantly higher WG, BWG, SGR and HPSI on a dry weight basis and increased moult frequency compared to 0% DM Chol (*P* < 0.05; Tukey’s test). Total bulk body weight of all lobsters in each replicate tank measured at 4 weeks revealed a ≥ twofold increase in average wet weight in all dietary treatments at this time, though weight gain was slightly lower for 0% DM Chol (Fig. 1). From this point, average weight decreased between weeks 4 and 8 for lobsters fed 0% DM Chol, while for lobsters fed ≥ 0.2% DM Chol, the average wet weight continued to increase relatively linearly. A significant Chol*PhosL interaction was observed for lobster survival (Table 1). Survival was significantly lower at 0% DM Chol compared to ≥ 0.2% DM dietary Chol in the absence of dietary PhosL (*P* < 0.05; Tukey’s test) but was similar for all Chol levels at 1% DM PhosL (*P* > 0.05; Tukey’s test). In this way, dietary PhosL significantly enhanced lobster survival at the lowest 0% DM dietary Chol level and slightly but significantly reduced survival at the highest 0.4% DM dietary Chol level. Significant effects of both dietary Chol and PhosL were found for AFI (Table 1). The AFI was significantly higher at dietary Chol ≥ 0.2% DM compared to 0% DM, but was simultaneously lower at each corresponding Chol level when combined with 1% DM PhosL (*P* < 0.05; Tukey’s test). The combined effects on growth and AFI resulted in equivalent differences in biological FCR at Chol ≥ 0.2% DM and additionally at each dietary Chol inclusion percentage when combined with 1% DM dietary PhosL (Table 1).

**Table 1:** Wet weight, dry weight and general growth performance parameters (mean ± SEM, n = 3) of juvenile *Thenus australiensis* at three cholesterol (Chol %) and two phospholipid (PhosL %) supplemental dietary concentrations after 8 weeks.

	Initial weight (g)	Final weight (g)	Daily WG (g d^−1^)	Total BWG (%)	SGR (%WG d^−1^)	HPSI (%)
Wet weight growth						
Chol level						
0.0%	4.49 ± 0.03	9.34 ± 0.20^a^	0.09 ± 0.00^a^	108.09 ± 4.43^a^	1.28 ± 0.04^a^	2.46 ± 0.12^a^
0.2%	4.50 ± 0.03	18.16 ± 0.68^b^	0.24 ± 0.01^b^	303.92 ± 14.34^b^	2.44 ± 0.06^b^	3.26 ± 0.10^b^
0.4%	4.52 ± 0.03	18.12 ± 0.91^b^	0.24 ± 0.02^b^	301.67 ± 21.26^b^	2.39 ± 0.09^b^	3.37 ± 0.11^b^
* P value*	0.751	** < 0.001**	** < 0.001**	** < 0.001**	** < 0.001**	** < 0.001**
PhosL level						
0.0%	4.46 ± 0.02	15.64 ± 1.60	0.20 ± 0.03	251.09 ± 35.77	2.12 ± 0.20	2.99 ± 0.15
1.0%	4.54 ± 0.02	14.77 ± 1.50	0.18 ± 0.03	224.69 ± 32.65	1.99 ± 0.19	3.04 ± 0.17
* P value*	0.152	0.200	0.136	0.072	0.066	0.766
Interaction (ChoL x PhosL)						
Chol 0.0%/PhosL 0.0%	4.46 ± 0.05	9.50 ± 0.21	0.09 ± 0.00	113.15 ± 5.03	1.33 ± 0.04	2.50 ± 0.23
Chol 0.2%/PhoL 0.0%	4.45 ± 0.03	17.97 ± 0.61	0.24 ± 0.01	304.00 ± 13.33	2.45 ± 0.06	3.36 ± 0.11
ChoL 0.4%/PhosL 0.0%	4.46 ± 0.02	19.47 ± 1.16	0.26 ± 0.02	336.13 ± 24.87	2.58 ± 0.10	3.12 ± 0.10
Chol 0.0%/PhosL 1.0%	4.52 ± 0.03	9.17 ± 0.36	0.08 ± 0.01	103.03 ± 6.89	1.24 ± 0.06	2.43 ± 0.15
Chol 0.2%/PhoL 1.0%	4.54 ± 0.05	18.36 ± 1.39	0.24 ± 0.02	303.84 ± 29.16	2.44 ± 0.12	3.17 ± 0.15
Chol 0.4%/PhoL 1.0%	4.57 ± 0.05	16.77 ± 0.97	0.21 ± 0.02	267.21 ± 21.32	2.28 ± 0.10	3.51 ± 0.11
* P value*	0.807	0.281	0.324	0.301	0.356	0.203
Dry weight growth						
Chol level						
0.0%	1.30 ± 0.01	2.28 ± 0.05^a^	0.02 ± 0.00^a^	75.63 ± 3.39^a^	0.99 ± 0.03^a^	1.88 ± 0.10^a^
0.2%	1.30 ± 0.01	4.48 ± 0.06^b^	0.06 ± 0.00^b^	244.78 ± 3.95^b^	2.17 ± 0.02^b^	4.21 ± 0.11^b^
0.4%	1.30 ± 0.01	4.50 ± 0.11^b^	0.06 ± 0.00^b^	245.20 ± 8.08^b^	2.17 ± 0.04^b^	4.15 ± 0.12^b^
P value	0.751	** < 0.001**	** < 0.001**	** < 0.001**	** < 0.001**	** < 0.001**
PhosL level						
0.0%	1.29 ± 0.00	3.78 ± 0.37	0.04 ± 0.01	193.79 ± 28.76	1.81 ± 0.20	3.52 ± 0.41
1.0%	1.31 ± 0.01	3.81 ± 0.40	0.04 ± 0.01	190.12 ± 30.11	1.78 ± 0.21	3.34 ± 0.38
* P value*	0.152	0.997	0.539	0.241	0.182	0.247
Interaction (ChoL x PhosL)						
Chol 0.0%/PhosL 0.0%	1.29 ± 0.01	2.33 ± 0.09	0.02 ± 0.00	80.55 ± 5.70	1.30 ± 0.05	1.91 ± 0.21
Chol 0.2%/PhoL 0.0%	1.28 ± 0.01	4.39 ± 0.05	0.05 ± 0.00	242.19 ± 6.56	2.16 ± 0.03	4.33 ± 0.11
ChoL 0.4%/PhosL 0.0%	1.29 ± 0.00	4.62 ± 0.16	0.06 ± 0.00	258.65 ± 12.79	2.24 ± 0.06	4.33 ± 0.11
Chol 0.0%/PhosL 1.0%	1.30 ± 0.01	2.23 ± 0.02	0.02 ± 0.00	70.72 ± 0.81	0.94 ± 0.01	1.85 ± 0.09
Chol 0.2%/PhoL 1.0%	1.31 ± 0.01	4.56 ± 0.09	0.06 ± 0.00	247.37 ± 5.32	2.18 ± 0.03	4.10 ± 0.19
Chol 0.4%/PhoL 1.0%	1.32 ± 0.01	4.65 ± 0.19	0.06 ± 0.00	252.29 ± 12.37	2.21 ± 0.06	4.06 ± 0.21
* P value*	0.807	0.423	0.254	0.251	0.176	0.859

General growth	Survival (%)	Moults (count)	Moult frequency (moults ind.^−1^)	AFI (mg DM ind.^−1^ d^−1^)	FCR (g feed g^−1^ dry WG)	
Chol level						
0.0%	58.33 ± 8.33^a^	10.33 ± 0.49^a^	1.41 ± 0.06^a^	68.54 ± 10.37^a^	3.94 ± 0.52^b^	
0.2%	95.83 ± 2.64^b^	17.83 ± 0.48^b^	2.29 ± 0.05^b^	123.60 ± 5.70^b^	2.22 ± 0.12^a^	
0.4%	89.58 ± 3.82^b^	17.5 ± 0.52^b^	2.24 ± 0.07^b^	122.76 ± 8.94^b^	2.19 ± 0.15^a^	
* P value*	** < 0.001**	** < 0.001**	** < 0.001**	** < 0.001**	** < 0.001**	
PhosL level						
0.0%	80.56 ± 9.11	15.56 ± 1.43	2.01 ± 0.16	122.61 ± 8.63^b^	3.30 ± 0.44^b^	
1.0%	81.94 ± 4.71	14.56 ± 1.02	1.90 ± 0.13	88.25 ± 10.66^a^	2.23 ± 0.20^a^	
* P value*	0.584	0.115	0.245	** < 0.001**	** < 0.001**	
Interaction (ChoL x PhosL)						
Chol 0.0%/PhosL 0.0%	45.83 ± 8.33^a^	10.00 ± 1.00	1.39 ± 0.13	89.91 ± 7.65	4.97 ± 0.46	
Chol 0.2%/PhoL 0.0%	100.00 ± 0.00^c^	18.67 ± 0.33	2.33 ± 0.04	135.74 ± 3.35	2.49 ± 0.02	
ChoL 0.4%/PhosL 0.0%	95.83 ± 4.17^c^	18.00 ± 0.00	2.32 ± 0.07	142.19 ± 3.40	2.44 ± 0.11	
Chol 0.0%/PhosL 1.0%	70.83 ± 11.02^b^	10.67 ± 0.33	1.44 ± 0.03	47.16 ± 4.76	2.92 ± 0.31	
Chol 0.2%/PhoL 1.0%	91.67 ± 4.17^bc^	17.00 ± 0.58	2.24 ± 0.10	111.46 ± 2.00	1.96 ± 0.08	
Chol 0.4%/PhoL 1.0%	83.33 ± 4.17^b^	16.00 ± 0.58	2.04 ± 0.05	106.14 ± 7.94	1.81 ± 0.06	
* P value*	**0.025**	0.093	0.216	0.255	0.093	

P value in bold indicate statistically significant difference (*P* < 0.05). . Where a statistical main effect (Chol and PhosL) or interaction (Chol*PhosL) was observed, superscripts are used to denote homogenous data ranges (*P* > 0.05) for treatment means determined by Tukey’s post-hoc analysis.

n.s. = not significant.

WG = weight gain; BWG = body weight gain; SGR = specific growth rate; HPSI = hepatosomatic index; AFI = apparent feed intake; DM = dry matter; FCR = feed conversion ratio.

A significant dietary Chol effect was found for all measures of calculated whole-body chemical composition except for DM (Table 2). Dietary Chol ≥ 0.2% DM resulted in significantly higher whole-body crude protein (CP), total lipid (TL), gross energy (GE), triacylglycerol (TAG) and total polar lipid (TPL) content and lower ash and total sterols (ST) content compared to those at 0% DM Chol (*P* < 0.05; Tukey’s test). Except for CP and ash, whole-body composition was similar between 0.2 and 0.4% DM Chol. In contrast, there was a significant trend of increasing CP and decreasing ash contents with increasing Chol. There were no significant treatment effects on tail muscle (TM) chemical composition, except for GE content where there was a significant dietary Chol effect (Table 3). Tail muscle GE content was higher at ≥ 0.2% DM Chol compared to 0% DM Chol (*P* < 0.05; Tukey’s test). A slightly more complex pattern of effects was found for HP chemical composition (Table 4). A significant dietary Chol effect was observed for DM, CP, ash, GE and sterol contents, where DM and GE content were higher while CP, ash and sterol content were lower at Chol ≥ 0.2% DM compared to 0% DM Chol (*P* < 0.05; Tukey’s test). For TL, TAG and TPL, a significant dietary Chol*PhosL interaction was found. Here there was a trend of increasing TL and TAG amounts with increasing Chol in the absence of dietary PhosL. At 1% DM PhosL, TL and TAG amounts were elevated at dietary Chol ≥ 0.2% DM compared to 0% DM Chol, but slightly lower at 0.4% compared to 0.2% DM Chol. For TPL, the inverse relationship was observed.

**Table 2:** Chemical composition and lipid profiles calculated for the whole-body (mean ± SEM, n = 3) of juvenile *Thenus australiensis* at three cholesterol (Chol, %) and two phospholipid (PhosL, %) supplemental dietary concentrations after 8 weeks.

	DM (% WW)	CP (% DM)	TL (% DM)	Ash (% DM)	GE (MJ kg^−1^ DM)	TAG (% TL)	ST (% TL)	TPL (% TL)
Chol level								
0.0%	24.44 ± 0.69	38.49 ± 0.51^a^	4.31 ± 0.38^a^	48.05 ± 1.08^b^	10.03 ± 0.25^a^	4.91 ± 0.66^a^	17.09 ± 1.04^b^	78.00 ± 1.35^b^
0.2%	24.78 ± 0.73	43.05 ± 0.84^b^	7.50 ± 0.24^b^	37.59 ± 0.71^a^	13.52 ± 0.12^b^	28.16 ± 1.32^b^	10.29 ± 0.56^a^	61.55 ± 1.78^a^
0.4%	25.84 ± 1.16	47.06 ± 0.92^b^	7.08 ± 0.33^b^	35.12 ± 0.87^a^	13.97 ± 0.20^b^	31.04 ± 2.57^b^	11.61 ± 0.91^a^	57.35 ± 2.10^a^
P value	0.476	** < 0.001**	** < 0.001**	** < 0.001**	** < 0.001**	** < 0.001**	** < 0.001**	** < 0.001**
PhosL level								
0.0%	24.3 ± 0.58	42.24 ± 1.28	6.57 ± 0.57	40.68 ± 2.06	12.42 ± 0.64	21.72 ± 4.56	12.86 ± 1.05	65.42 ± 3.72
1.0%	25.74 ± 0.78	43.49 ± 1.44	6.02 ± 0.53	39.83 ± 2.14	12.59 ± 0.64	21.02 ± 4.13	13.14 ± 1.41	65.84 ± 3.15
*P value*	0.152	0.195	0.213	0.463	0.522	0.912	0.871	0.871
Interaction (ChoL x PhosL)								
Chol 0.0%/PhosL 0.0%	24.53 ± 1.25	38.16 ± 1.08	4.50 ± 0.69	48.41 ± 1.58	9.93 ± 0.34	4.19 ± 0.66	16.63 ± 0.42	79.18 ± 0.27
Chol 0.2%/PhoL 0.0%	24.52 ± 0.80	41.91 ± 0.42	7.66 ± 0.50	38.03 ± 0.18	13.45 ± 0.13	26.61 ± 0.56	10.09 ± 0.56	63.29 ± 0.46
ChoL 0.4%/PhosL 0.0%	23.85 ± 1.01	46.65 ± 0.47	7.54 ± 0.50	35.59 ± 1.40	13.90 ± 0.28	34.34 ± 1.89	11.86 ± 1.11	53.80 ± 1.05
Chol 0.0%/PhosL 1.0%	24.34 ± 0.58	38.82 ± 0.15	4.11 ± 0.45	47.69 ± 1.79	10.13 ± 0.44	5.62 ± 1.11	17.56 ± 2.23	76.82 ± 2.78
Chol 0.2%/PhoL 1.0%	25.04 ± 1.21	44.18 ± 1.44	7.34 ± 0.49	37.14 ± 1.52	13.59 ± 0.21	29.70 ± 2.44	10.49 ± 1.11	59.81 ± 3.55
Chol 0.4%/PhoL 1.0%	27.83 ± 1.16	47.46 ± 1.95	6.62 ± 0.27	34.65 ± 1.26	14.05 ± 0.34	27.74 ± 4.30	11.37 ± 1.68	60.89 ± 2.89
*P value*	0.197	0.736	0.847	0.995	0.992	0.111	0.882	0.082

TPL = combined phospholipids, sphingolipids, glycolipids, carotenoid pigments and residual non-lipid material.

P value in bold indicate statistically significant difference (*P* < 0.05). Where a statistical main effect (Chol and PhosL) or interaction (Chol*PhosL) was observed, superscripts are used to denote homogenous data ranges (*P* > 0.05) for treatment means determined by Tukey’s post-hoc analysis.

*n.s.* not significant.

*DM* dry matter, *WW* wet weight, *CP* crude protein, *TL* total lipid, *GE* gross energy, *TAG* triacylglycerol, *ST* total sterols, *TPL* total polar lipid.

**Table 3:** Chemical composition and lipid profiles in tail muscle tissue (mean ± SEM, n = 3) of juvenile *Thenus australiensis* at three cholesterol (Chol, %) and two phospholipid (PhosL, %) supplemental dietary concentrations after 8 weeks.

	DM (% WW)	CP (% DM)	TL (% DM)	Ash (% DM)	GE (MJ kg^−1^ DM)	TAG (% TL)	ST (% TL)	TPL (% TL)
Chol level								
0.0%	18.02 ± 0.49	86.63 ± 0.92	7.04 ± 0.58	9.52 ± 0.20	18.04 ± 0.18^a^	n.d	23.31 ± 1.47	76.69 ± 1.47
0.2%	19.81 ± 0.35	85.14 ± 0.52	7.98 ± 0.55	9.35 ± 0.29	19.63 ± 0.19^b^	n.d	20.93 ± 0.83	79.07 ± 0.83
0.4%	20.6 ± 1.04	86.01 ± 1.03	7.54 ± 1.13	8.82 ± 0.16	19.92 ± 0.07^b^	n.d	21.80 ± 1.30	78.2 ± 1.30
*P value*	0.065	0.472	0.675	0.158	** < 0.001**		0.326	0.326
PhosL level								
0.0%	19.04 ± 0.48	85.09 ± 0.55	8.25 ± 0.69	9.28 ± 0.25	19.20 ± 0.35	n.d	21.52 ± 1.03	78.48 ± 1.03
1.0%	19.90 ± 0.79	86.8 ± 0.71	6.79 ± 0.47	9.18 ± 0.15	19.19 ± 0.28	n.d	22.51 ± 1.00	77.49 ± 1.00
*P value*	0.322	0.097	0.135	0.748	0.994		0.431	0.431
Interaction (ChoL × PhosL)								
Chol 0.0%/PhosL 0.0%	17.77 ± 1.00	85.92 ± 1.53	6.80 ± 1.19	9.65 ± 0.42	17.86 ± 0.32	n.d	25.07 ± 0.89	74.93 ± 0.89
Chol 0.2%/PhoL 0.0%	19.79 ± 0.32	84.79 ± 0.88	9.11 ± 0.13	9.40 ± 0.55	19.89 ± 0.14	n.d	20.12 ± 0.65	79.88 ± 0.65
ChoL 0.4%/PhosL 0.0%	19.55 ± 0.64	84.56 ± 0.04	8.82 ± 1.63	8.80 ± 0.28	19.84 ± 0.12	n.d	19.37 ± 1.40	80.63 ± 1.40
Chol 0.0%/PhosL 1.0%	18.26 ± 0.41	87.35 ± 1.16	7.27 ± 0.42	9.40 ± 0.08	18.22 ± 0.17	n.d	21.55 ± 2.63	78.45 ± 2.63
Chol 0.2%/PhoL 1.0%	19.83 ± 0.72	85.58 ± 0.66	6.84 ± 0.47	9.30 ± 0.34	19.37 ± 0.30	n.d	21.75 ± 1.53	78.25 ± 1.53
Chol 0.4%/PhoL 1.0%	21.62 ± 1.99	87.46 ± 1.80	6.25 ± 1.43	8.85 ± 0.24	19.99 ± 0.06	n.d	24.23 ± 0.76	75.77 ± 0.76
*P value*	0.612	0.631	0.309	0.924	0.125		0.144	0.144

TPL = combined phospholipids, sphingolipids, glycolipids, carotenoid pigments and residual non-lipid material.

P value in bold indicate statistically significant difference (*P* < 0.05). Where a statistical main effect (Chol and PhosL) or interaction (Chol*PhosL) was observed, superscripts are used to denote homogenous data ranges (*P* > 0.05) for treatment means determined by Tukey’s post-hoc analysis.

n.s. = not significant; n.d. = not detected.

*DM* dry matter, *WW* wet weight, *CP* crude protein, *TL* total lipid, *GE* gross energy, *TAG* triacylglycerol, *TPL* total polar lipid.

**Table 4:** Chemical composition and lipid profiles in hepatopancreas tissue (mean ± SEM, n = 3) of juvenile *Thenus australiensis* at three cholesterol (Chol, %) and two phospholipid (PhosL, %) supplemental dietary concentrations after 8 weeks.

	DM (% WW)	CP (% DM)	TL (% DM)	Ash (% DM)	GE (MJ kg^−1^ DM)	TAG (% TL)	ST (% TL)	TPL (% TL)
Chol level								
0.0%	18.63 ± 0.61^a^	55.43 ± 1.05^b^	21.16 ± 0.66^a^	10.26 ± 0.80^b^	19.84 ± 0.67^a^	43.68 ± 2.25	3.45 ± 0.28b	52.87 ± 2.00^b^
0.2%	31.96 ± 0.62^b^	33.36 ± 1.02^a^	51.88 ± 1.99^b^	6.10 ± 0.28^a^	27.08 ± 0.33^b^	87.70 ± 0.92	0.35 ± 0.05a	11.95 ± 0.96^a^
0.4%	32.64 ± 0.78^b^	31.00 ± 1.68^a^	54.60 ± 2.83^b^	5.67 ± 0.27^a^	27.88 ± 0.33^b^	86.66 ± 2.27	0.19 ± 0.06a	13.15 ± 2.25^a^
P value	** < 0.001**	** < 0.001**	** < 0.001**	** < 0.001**	** < 0.001**	** < 0.001**	** < 0.001**	** < 0.001**
PhosL level								
0.0%	27.78 ± 2.30	40.04 ± 4.34	42.31 ± 5.71	7.28 ± 0.93	24.43 ± 1.42	73.39 ± 8.73b	1.54 ± 0.63	25.08 ± 8.10a
1.0%	27.71 ± 2.35	39.81 ± 3.78	42.78 ± 5.45	7.41 ± 0.76	25.43 ± 1.18	71.97 ± 6.06a	1.12 ± 0.46	26.91 ± 5.61b
* P value*	0.931	0.893	0.756	0.766	0.064	**0.036**	0.145	**0.016**
Interaction (ChoL × PhosL)								
Chol 0.0%/PhosL 0.0%	18.65 ± 1.06	56.65 ± 1.33	20.23 ± 0.85^a^	10.22 ± 1.74	18.83 ± 0.73	39.40 ± 1.64^a^	3.98 ± 0.05	56.63 ± 1.62^b^
Chol 0.2%/PhoL 0.0%	31.57 ± 0.26	34.29 ± 0.58	48.13 ± 0.88^b^	6.34 ± 0.13	26.82 ± 0.58	89.48 ± 0.36^c^	0.44 ± 0.08	10.08 ± 0.37^a^
ChoL 0.4%/PhosL 0.0%	33.12 ± 1.00	29.19 ± 0.95	58.58 ± 0.26^c^	5.26 ± 0.12	27.65 ± 0.66	91.29 ± 1.99^c^	0.19 ± 0.10	8.52 ± 1.94^a^
Chol 0.0%/PhosL 1.0%	18.61 ± 0.85	54.21 ± 1.50	22.09 ± 0.75^a^	10.30 ± 0.37	20.85 ± 0.82	47.97 ± 2.06^b^	2.91 ± 0.34	49.12 ± 1.79^c^
Chol 0.2%/PhoL 1.0%	32.36 ± 1.30	32.43 ± 2.01	55.63 ± 2.22^c^	5.86 ± 0.57	27.33 ± 0.38	85.91 ± 0.97^d^	0.27 ± 0.00	13.82 ± 0.97^d^
Chol 0.4%/PhoL 1.0%	32.16 ± 1.35	32.80 ± 3.15	50.61 ± 4.92^bc^	6.08 ± 0.43	28.12 ± 0.22	82.03 ± 0.52^e^	0.19 ± 0.10	17.78 ± 0.49^e^
*P value*	0.735	0.223	**0.017**	0.600	0.374	** < 0.001**	0.457	** < 0.001**

TPL = combined phospholipids, sphingolipids, glycolipids, carotenoid pigments and residual non-lipid material.

P value in bold indicate statistically significant difference (*P* < 0.05). Where a statistical main effect (Chol and PhosL) or interaction (Chol*PhosL) was observed, superscripts are used to denote homogenous data ranges (*P* > 0.05) for treatment means determined by Tukey’s post-hoc analysis.

*DM* dry matter, *WW* wet weight, *CP* crude protein, *TL* total lipid, *GE* gross energy, *TAG* triacylglycerol, *TPL* total polar lipid.

## Discussion

Cholesterol is considered an essential nutrient for decapod crustaceans, although the quantitative dietary requirements have been shown to vary for different species, developmental stages, life histories and in combination with other key nutrients^3^. Despite possessing key attributes desirable for experimentation and captive rearing^30,31^, slipper lobsters (Scyllaridae) remain a critically understudied decapod family. Consequently, little is known regarding the nutritional requirements of the slipper lobster, *T. australiensis*. While recent studies have started to explore their nutritional requirements and physiology in more detail^28,30,32,33^, the present study is the first to examine the importance of dietary Chol. For several crustacean species other than the slipper lobsters, there are some evidence that dietary PhosL may spare Chol^3^. Thus, in the present study, such interactive effects were investigated. It was found that provision of sufficient dietary Chol (between 0.05 and 0.2% DM in feed) is essential for normal moulting and growth. Cholesterol was the sole factor linked to significant differences in growth and general nutritional condition (whole-body) at dietary Chol levels ≥ 0.2% DM, while 1% DM dietary PhosL had significant effects on AFI and FCR resulting in improved feed efficiency. Furthermore, an interactive effect between dietary Chol and PhosL was found for lobster survival, primarily whereby inclusion of dietary PhosL significantly enhanced survival at the lowest dietary Chol level.

Moulting of juvenile *T. australiensis* was initially similar for all dietary treatments over the first 2–3 weeks of the experiment, generally until after the first moult. At 4 weeks, subsequent moulting had already commenced for some lobsters in dietary treatments with ≥ 0.2% DM dietary Chol, seemingly accounting for slight differences in average wet weight of lobsters between 0% and ≥ 0.2% DM dietary Chol at 4 weeks. Over the remainder of the experiment, moulting slowed dramatically at 0% DM dietary Chol but continued as expected at ≥ 0.2% DM dietary Chol. These observations are consistent with previous findings that dietary Chol can influence moult frequency, intermoult duration and development time in mud crab^13^, Chinese mitten crab, *Eriocheir sinensis*^15^ and blue swimmer crab, *Portunus pelagicus*^12^. The moult cycle is a fundamental aspect of crustacean biology that is intimately linked with metamorphosis, growth and reproduction^30,34–36^. Since Chol is the principal precursor for the ecdysteroid moult hormones^4^, dietary Chol is expected to play an essential role in the regulation of moulting.

Survival was also significantly affected at 0% DM Chol with most mortalities occurring after the first moult. However, survival was significantly improved at 0% DM dietary Chol (45.83 ± 8.33%) when combined with 1% DM dietary PhosL (70.83 ± 11.02%). Studies have shown that insufficient dietary Chol can negatively affect survival and has been linked to a condition termed ‘moult death syndrome’ (incomplete ecdysis) in homarid lobsters *Homarus* sp.^37^, crayfish *Pacifastacus leniusculus*^16^ and crabs^12^. The present study similarly demonstrated that 0% DM dietary Chol disrupted the moult cycle of juvenile *T. australiensis*, although moult failure (incomplete ecdysis) was not observed, and in turn affected growth and survival rates. Since effects generally did not manifest at 0% DM dietary Chol until after the first moult, there is an implied connection between dietary intake/ availability and changes in nutritional condition over time.

Experimental feed Chol measured by GC–MS closely matched the target inclusion amounts of 0.2 and 0.4% DM, while a trace amount of dietary Chol of approx. 0.05% DM was measured in feeds without Chol supplementation (nominally 0% DM dietary Chol). It was clear that dietary Chol had highly significant effects on the growth of juvenile *T. australiensis*. There was no difference in growth responses between 0.2 and 0.4% DM dietary Chol or between 0 and 1% DM dietary PhosL at any dietary Chol amount. However, after 8 weeks overall growth responses were approximately two- to threefold greater at ≥ 0.2% DM dietary Chol compared to 0% DM dietary Chol. Furthermore, comparable feeding and growth was observed for all treatments through the first moult of the experiment, after which feeding reduced, and growth plateaued or decreased in lobsters at 0% DM dietary Chol. Therefore, the poor growth observed at 0% DM dietary Chol was related to differences in both AFI and feed utilisation and, in accordance with FCR, it was observed that the 0% DM dietary Chol juveniles effectively consumed more feed per unit of growth. The poor growth of juvenile lobsters at 0% DM dietary Chol meant that, in absolute terms, AFI was significantly lower than the 0.2 and 0.4% DM Chol supplemented treatments. The lower growth at 0% DM dietary Chol in relation to FCR and AFI lead us to conclude that these effects were driven by Chol deficiency and resulting disruptions in normal behaviour and/ or physiological processes (e.g. moulting).

Growth at ≥ 0.2% DM dietary Chol was comparable with recent studies using 0.5% DM supplemental Chol in experimental feed formulations, where much higher total sterols were also measured (up to 1.5% DM), and *T. australiensis* lobsters of similar juvenile stage and experimental duration^28,32^. The apparent Chol requirement for growth of juvenile *T. australiensis* is relatively consistent with that for other decapod crustaceans. For penaeid shrimp, dietary Chol requirements have been reported to vary between 0.2 and 2.1%^2^. This range is skewed at the higher end by the results of Deshimaru and Kuroki^38^ for the kuruma prawn, *Marsupenaeus japonicus*, although other early studies with this species also report wide-ranging optima of 0.2% to 1.0% supplemental Chol for larval and juvenile life stages^39–41^. Teshima et al.^42^ later refined the requirement for juvenile kuruma prawn to between approx. 0.29 and 0.60% actual Chol in the diet. Studies involving other species also report Chol requirements for optimal growth to be low, typically around ≤ 0.5% of the diet. For juvenile black tiger shrimp, *Penaeus monodon*, Chen^8^ reported optimum growth at 0.5% supplemental dietary Chol. Sheen et al.^43^ observed maximal growth at 0.19% actual dietary Chol with no significant difference in growth rate up to 0.81% in the diet of juvenile *P. monodon*. In line with these findings, Smith et al.^44^ determined the optimal supplemental Chol requirement for subadult *P. monodon* to be around 0.17% of the diet. Similar low Chol requirements have been found for the Pacific white shrimp, *L. vannamei*^23,26^. For Pacific white shrimp, the maximum growth rate of juveniles has been observed at 0.11% actual Chol in the diet with an optimum of 0.15% determined by regression analysis^26^. Similar findings have been made for the American lobster, *H. americanus*, where supplemental Chol requirements have been reported in the range of 0.12–0.25% for larval and juvenile stages^18,21^) with 0.5% achieving best growth rates in juveniles in another study^20^. For redclaw crayfish, *Cherax quadricarinatus*, an optimum of 0.49% actual dietary Chol was found^17^. Sheen^13^ determined an optimum of 0.51% dietary Chol by regression analysis in juvenile mud crab, *Scylla serrata*. Irvin et al.^22^ could not determine a dietary Chol requirement for juvenile tropical rock lobster, *Panulirus ornatus*, but suggested it was less than 0.125% of the diet.

Differences in Chol requirements between studies might be in part due to the presence of other sterols in feeds and the ability of certain species to effectively utilise these compounds^6,9,26^. In the present study, total sterols determined by TLC–FID were at least twofold greater than actual Chol measured by GC–MS due to unidentified sterol compounds present in the feed basal ingredients. Furthermore, since the presence of these sterols did not result in adequate growth or survival in the absence of supplemental dietary Chol, this may indicate juvenile *T. australiensis* have a specific requirement for Chol and/ or limited ability to effectively utilise other sterols. This may not be surprising given the predominately carnivorous and specialised feeding habits of *Thenus* spp.^45,46^.

Dietary PhosLs have previously been associated with Chol ‘sparing’ in some species^3^. While dietary PhosL did have significant effects on AFI and FCR, significant effects on the standard growth endpoints were not directly observed in juvenile *T. australiensis*. This could be at least in part due to the range of dietary Chol and PhosL concentrations examined. For example, Gong et al.^23^ found similar growth rates at 0.2 and 0.5% dietary Chol at all PhosL concentrations ranging from 0 to 5% in juvenile Pacific white shrimp. In a second experiment targeting a much lower dietary Chol range, they then observed a strong interactive effect between Chol and PhosL. In the second experiment, the Chol requirement for growth in juvenile Pacific white shrimp decreased significantly as dietary PhosL was serially increased, such that the Chol requirements were 0.05%, 0.13%, 0.14% and 0.35% at 5%, 3%, 1.5% and 0% total PhosL, respectively. Given the observed PhosL effects on AFI and FCR, and interactive effect on lobster survival, similar findings to Gong et al.^23,47^ may also be observed for juvenile *T. australiensis* if different dietary Chol and PhosL ranges are employed in future experimental research.

Dietary Chol was the most significant factor in determining chemical composition and overall nutritional condition in juvenile *T. australiensis*. At ≥ 0.2% DM dietary Chol, whole-body composition was broadly consistent with that expected for juvenile *T. australiensis* over the moult^30^. However, at 0% DM dietary Chol, lobsters were found to be in poor nutritional condition at the end of the experiment, as indicated by lower whole-body CP, TL, GE and higher proportional ash content. Whole-body lipid class profiles also revealed significantly reduced TAG and higher proportional ST and TPL contents, driven almost entirely by effects on HPSI and HP lipid content. The overall Chol*PhosL interaction for TL, TAG and TPL content of the HP is not easily explained. When viewed alongside moulting data, minor differences might be associated with slight variations in moult stage and timing between treatments^30^. However, these relatively minor differences aside, the greatest differences in HP lipid content and composition were very clearly associated with 0% DM dietary Chol. Lipid and energy storage is particularly important in crustaceans to endure transient non-feeding stages^48,49^ and the energy demanding metabolic processes associated with ecdysis^34,50,51^. Reduced feed intake and mass loss between weeks 4 and 8, after the first moult had been successfully completed, suggests that at 0% DM dietary Chol juvenile *T. australiensis* had become dependent on utilisation of tissues to meet metabolic requirements^52^ and after 8 weeks had virtually exhausted their HP TAG energy reserves. This starvation-like state and apparent inability to successfully complete a second moult *en masse* likely intensified effects of dietary Chol deficiency on moulting and perhaps ultimately affected survival. Although survival was higher at 0% DM dietary Chol when combined with 1% DM dietary PhosL, whole-body composition was similar at both dietary PhosL levels and all surviving lobsters were in similarly poor nutritional condition at the conclusion of the experiment. Tail muscle chemical composition did not differ between dietary treatments except for GE content which was significantly lower at 0% DM dietary Chol. In Atlantic ghost crab, *Ocypode quadrata*, 15-d starvation was found to have different metabolic effects in the different tissues observed as HP lipolysis and β-oxidation, HP gluconeogenesis and muscle glycogenolysis with depletion of glycogen^53^. Therefore, reduced feed intake resulting in the requirement for muscle glycogen as energy offers one possible explanation for the lower GE content in the TM. Nevertheless, the lack of other significant differences suggests that TM chemical composition may not be a particularly useful indicator of nutritional condition for *T. australiensis*. Low CP and high ash content of the whole-body indicates that lobsters at 0% DM dietary Chol likely possessed reduced total muscle content. Musculosomatic index (MSI) has previously been shown can decrease significantly following starvation in black tiger shrimp^54^ and so in future MSI may be a more useful supporting indicator of nutritional condition in juvenile *T. australiensis*.

The lack of clear significant effects of dietary PhosL on juvenile *T. australiensis* growth endpoints, probable indirect effects from improved feed efficiency aside, is worthy of some further consideration. In general, dietary PhosLs are also regarded as essential nutrients for fish and crustaceans^27^, though this is not as well-established in the literature for crustaceans^28^. Recent research has shown that dietary phosphatidylcholine (PC) is probably not essential for later stage juvenile *T. australiensis* (juvenile instar J6 onwards) owing to an apparent capacity for endogenous biosynthesis far exceeding possible dietary uptake^28^. Earlier stage juveniles (juvenile instar J4 onwards) were selected for the current experiment on the assumption that PhosL biosynthetic capacity may improve during juvenile development and theoretically reduced lipid digestive and biosynthetic capacities in earlier juveniles might help to uncover dietary nutrient relationships^27,55^. Supplemental choline was also omitted from experimental feed formulations, previously administered at 0.6% DM choline chloride^28^, to further limit the potential involvement of endogenous PC biosynthesis during growth. However, there were still trace amounts of choline found in all feeds, primarily derived from ingredients in the basal mix (approx. 0.25% DM) with an additional smaller contribution (approx. 0.05%) from refined soy lecithin in the 1% DM PhosL supplemented feeds. Therefore, endogenous PC synthesis in the absence of supplemental dietary PhosL cannot be ruled out in this experiment as having a potential effect on dietary Chol uptake and mobilisation. However, Gong et al.^47^ previously did not observe an interaction between dietary Chol and PC in juvenile Pacific white shrimp, indicating that Chol ‘sparing’ in this species may be associated with PhosL’s other than PC^23^. Despite the inclusion of mixed PhosL’s in the form of refined soy lecithin, 1% DM supplemental PhosL resulted in fewer effects on overall growth performance compared to dietary Chol alone in the present study, potentially providing additional evidence that dietary PhosL requirements may be relatively low for juvenile *T. australiensis*^28^. However, inclusion of 1% DM supplemental dietary PhosL did have a significant beneficial effect on feed intake and feed conversion whereby less feed was consumed for each dietary Chol whilst achieving equivalent growth rates. Dietary PhosL at 1% DM also significantly improved lobster survival at the lowest dietary Chol level of 0% DM. Together these findings demonstrate that dietary PhosL had significant beneficial effects on nutrient uptake and/ or utilisation which may be expected given that dietary PhosL has been shown to support the mobilisation and transport of lipids from the gut into the haemolymph through Chol phospholipid lipoprotein complexes^37,56–62^. Although dietary PhosL effects in this study were not as pronounced as those for dietary Chol, these effects are nonetheless important and may have particular value for practical feed development and commercial applications.

## Conclusions

This study confirms that dietary Chol is essential for juvenile *T. australiensis*. After 8 weeks, lobsters fed 0% DM dietary Chol had significantly lower feed intake, moult frequency, growth, survival and nutritional condition compared to those at ≥ 0.2% DM dietary Chol. Cholesterol was the sole factor linked to differences in growth rates and whole-body chemical composition (condition) between 0% and ≥ 0.2% DM dietary Chol treatments, while dietary PhosL had a significant beneficial effect on feed efficiency (AFI and FCR) at each dietary Chol level. A significant interactive effect (Chol*PhosL) was also found for lobster survival, primarily whereby 1% DM dietary PhosL benefited survival at the lowest 0% DM dietary Chol level. This study confirms the essentiality of dietary Chol for juvenile *T. australiensis*, that actual dietary Chol requirements appear to be relatively low at ≤ 0.2% DM and that higher dietary Chol may not provide any additional benefit to direct growth or condition. However, juvenile *T. australiensis* may have a specific dietary requirement for Chol and/ or limited ability to effectively utilise other sterols. The findings of a generally low dietary Chol requirement coupled with improved feed efficiency linked to dietary PhosL have significant value for future commercial lobster farming for waste minimisation and potential economic sustainability.

## Methods

### Animals

Lobster husbandry and experimental research was conducted within the general framework and principles of the Australian Code for the Care and Use of Animals for Scientific Purposes^63^. The University of Tasmania does not require Animal Ethics Committee approval for research conducted on crustaceans based on the Tasmanian Animal Welfare Act 1993 (https://www.legislation.tas.gov.au/view/whole/html/inforce/current/act-1993-063), which stipulates that Animal Ethics Committee approval is required for animal research conducted on living, non-human vertebrates and cephalopods.

Juvenile slipper lobsters (*Thenus australiensis*) were reared from egg at the University of Tasmania, Institute for Marine and Antarctic Studies (IMAS), in February 2021 according to previously established protocols for tropical rock lobsters^64,65^. Post-hatchery juvenile slipper lobsters were cultured on fresh bivalve (*Plebidonax deltoides*) gonad until the third juvenile instar (J3) and then weaned onto a commercial-in-confidence nursery feed until intermoult J4 prior to experimentation. Upon commencement of the experiment, lobsters were individually weighed and then allocated to experimental aquaria based on size to ensure uniformity across all experimental treatment replicates (mean ± SD 4.50 ± 0.72 g, CV = 0.16). A total of 144 lobsters were stocked in 18 individual 18 L aquaria (0.38 m length × 0.24 m width × 0.25 m height) at a density of approx. 88 lobsters m^−2^ (n = 8 lobsters per aquarium). At the time of stocking, an additional 8 lobsters were weighed (mean ± SD 4.13 ± 1.08 g, CV = 0.26), induced into a cold coma by emersion in a seawater ice slurry for 4–5 min, dissected and carcass remains/ tissues stored frozen at − 80 °C for baseline chemical composition^28,32^. Situated within a recirculating aquaculture system (RAS), all experimental aquaria were supplied with filtered, ozonated seawater at 27 °C at a rate of 6 exchanges h^−1^ and maintained under a 12:12 h blue light: dark photoperiod. Water quality (dissolved oxygen, salinity, pH, ORP and temperature) was monitored and recorded daily.

### Experimental feeds

Six experimental feeds (Table 5), using a commercial-in-confidence basal mix, were prepared using three supplemental Chol concentrations of 0, 0.2 and 0.4% DM at two different supplemental phospholipid concentrations of 0 and 1.0% DM. To minimise potential spoilage and maintain freshness, experimental feeds were produced fortnightly using freshly prepared basal mix. Each feed was prepared by weighing out the required amount of fixed dry ingredient comprising the basal mix into a bowl. Lipid ingredients (fish oil, refined soy lecithin and Chol) were individually weighed out and combined to form a homogeneous lipid mixture. The lipid mixture was then slowly added to the basal mix and blended to a homogeneous semi-dry mash. The required water content was then added to the mash prior to feed manufacture. Details of the feed manufacturing method are commercial-in-confidence. After feed manufacture, a subsample (approx. 6 g fresh weight) was collected from each batch of fresh, dried feed (n = 4 per feed) and stored frozen at − 20 °C pending chemical analysis (Table 6). Working feeds were stored at 4 °C in sealed, air-tight containers between fortnightly feed production batches, after which remaining feeds were discarded.

**Table 5:** Ingredient composition (% dry matter) of experimental feeds consisting of three cholesterol (Chol %) and two phospholipid (PhosL %) concentrations.

Ingredient	Experimental feed					
	PhosL 0%			PhosL 1.0%		
	Chol 0%	Chol 0.2%	Chol 0.4%	Chol 0%	Chol 0.2%	Chol 0.4%
Basal mix^1^	92.0	92.0	92.0	92.0	92.0	92.0
Refined Soy Lecithin^2^	0	0	0	1.65	1.65	1.65
Purified fish oil^3^	8	7.8	7.6	6.35	6.15	5.95
Chol^4^	0	0.2	0.4	0	0.2	0.4

^1^Basal mix—Commercial in confidence.

^2^Ultralec® P Deoiled Lecithin—PhosL = 60% (Product 700851—ADM Australia Pty. Ltd).

^3^From menhaden (Product F8020—Sigma Aldrich, Castle Hill, NSW, Australia).

^4^ ≥ 99% Chol (Product C8667, Sigma Aldrich, Castle Hill, NSW, Australia).

**Table 6:** Chemical and lipid class composition data (mean ± SD, n = 2) for homogenised composite experimental feeds comprised of three cholesterol (Chol, %) and two phospholipid (PhosL, %) concentrations. Experimental feeds were subsampled fortnightly (n = 4) to reflect average feeds composition over the duration of the experiment and then analysed in duplicate.

	Experimental feed					
	PhosL 0%			PhosL 1.0%		
	Chol 0%	Chol 0.2%	Chol 0.4%	Chol 0%	Chol 0.2%	Chol 0.4%
Fresh Feed DM (% WW)	92.99 ± 0.26	93.25 ± 0.14	93.35 ± 0.43	93.77 ± 0.36	93.48 ± 0.16	93.59 ± 0.15
CP (% DM)	62.53 ± 1.89	62.69 ± 1.38	62.12 ± 5.83	60.96 ± 3.62	62.46 ± 2.12	61.35 ± 3.58
TL (% DM)	8.98 ± 0.06	9.08 ± 0.01	9.26 ± 0.05	8.81 ± 0.10	9.09 ± 0.14	8.82 ± 0.22
Ash (% DM)	9.37 ± 0.00	9.26 ± 0.00	9.31 ± 0.00	9.39 ± 0.00	9.29 ± 0.00	9.16 ± 0.00
Total Choline (% DM)	0.25 ± 0.01	0.26 ± 0.02	0.26 ± 0.00	0.30 ± 0.05	0.32 ± 0.07	0.30 ± 0.06
NFE (% DM)	18.88 ± 1.96	18.71 ± 1.39	19.05 ± 5.78	20.55 ± 3.68	18.83 ± 2.04	20.37 ± 3.74
GE (MJ kg^−1^ DM)	22.12 ± 0.13	22.18 ± 0.15	22.29 ± 0.09	22.02 ± 0.07	22.07 ± 0.09	22.08 ± 0.06
TAG (% DM)	7.36 ± 0.28	7.29 ± 0.12	7.17 ± 0.05	6.41 ± 0.07	6.25 ± 0.09	5.64 ± 0.12
ST (% DM)	0.21 ± 0.03	0.47 ± 0.04	0.79 ± 0.01	0.17 ± 0.04	0.42 ± 0.05	0.66 ± 0.11
Cholesterol (% DM)	0.06 ± 0.00	0.21 ± 0.00	0.39 ± 0.06	0.05 ± 0.00	0.20 ± 0.01	0.35 ± 0.01
TPL (% DM)	1.41 ± 0.25	1.33 ± 0.16	1.30 ± 0.04	2.23 ± 0.03	2.42 ± 0.04	2.52 ± 0.23

TPL = combined phospholipids, sphingolipids, glycolipids, carotenoid pigments and residual non-lipid material.

*DM* dry matter, *WW* wet weight, *CP* crude protein, *TL* total lipid, *NFE* nitrogen free extract, *GE* gross energy, *TAG* triacylglycerol, *ST* total sterols, *TPL* total polar lipid.

Justification of the chosen experimental feed lipid ingredient type and levels is provided as follows. In general, ingredient supplement levels were chosen based on previous similar works for other crustacean species^5,22,23,47,66^. As already described, Chol requirements for a broad range of decapod species has been found to be relatively low, generally ≤ 0.5% of the diet, and higher inclusion levels of > 0.8% DM can lead to negative effects in some species. However, Chol essentiality has not previously been established for any scyllarid or palinurid lobsters. Previous experimental studies on juvenile *T. australiensis* have employed experimental feeds with 0.5% DM supplemental Chol and measured total sterol levels ranging from 0.59 to 1.50% DM, without obvious negative effects or nutritional deficiencies being observed between feed treatments and experiments^28,32^. Landman et al.^32^ also included a natural, fresh feed treatment of blue mussel (*Mytilus galloprovincialis*) possessing approx. 0.9% DM total sterols. Within the related palinurid lobsters, Irvin et al.^22^ attempted to determine dietary Chol requirements for juvenile tropical rock lobsters (*Panulirus ornatus*) but were unable to confirm essentiality or a definitive requirement in the absence of differing dietary Chol effects between 0.125 and 0.425% DM. For these reasons, supplemental Chol inclusion percentages of 0, 0.2 and 0.4% DM were selected for the current experiment to examine dietary Chol essentiality in juvenile *T. australienis*. Research has also shown that dietary phosphatidylcholine (PC) is probably not essential for juvenile *T. australiensis* owing to an apparent capacity for endogenous biosynthesis far exceeding possible dietary uptake^28^. Similarly, Gong et al.^47^ also did not observe an interaction between dietary Chol and PC in juvenile Pacific white shrimp, indicating that Chol ‘sparing’ in this species may be associated with PhosL’s other than PC^23^. For these reasons, a mixed PhosL source from refined soy lecithin at inclusion percentages of 0% and 1.0% DM was chosen to explore possible interactions of dietary PhosL and Chol in case other dietary PhosLs are also functionally more important than PC in juvenile *T. australiensis*. To mitigate potential effects of fatty acid composition from the plant based PhosL source (soy lecithin), an ultra-pure marine fish oil was selected to balance the total lipid content of feeds (isolipidic) and the ensure provision of essential fatty acids.

### Experimental design

This experiment employed a 3 × 2 factorial design using the six experimental feeds as described above. Triplicate feed treatments were randomly allocated to experimental aquaria with the experiment conducted over eight consecutive weeks. The feeding protocol is described in the following section. Survival and moulting were monitored and recorded daily. Mortalities and exuvia were removed and visually inspected twice daily for evidence of disease or cannibalism. On completion of the experiment, surviving lobsters (total n = 117) were anaesthetised by submerging in a slurry of ice and seawater for 4–5 min. Each lobster was then individually blotted dry by paper towel before recording weight, carapace width and length measurements^30^. Each lobster was carefully dissected to remove the whole hepatopancreas (HP) and approximately 2 × 1 cm (length × width) strips of tail muscle (TM) tissue. Tissue samples were weighed for HPSI and subsequent biochemistry calculations. Following measurement and dissection, the carcass remains, HP and TM tissue samples were individually stored frozen at − 80 °C in preparation for freeze-drying and chemical analysis. Tail muscle, HP and carcass remains were analysed separately and the combined results used to calculate whole-body composition.

### Feeding protocol and apparent feed intake

Experimental feeds were supplied in excess of requirements continuously over 16 h day^−1^ (approximately 1700 to 0900 h daily) using belt feeders, with a daily ration of approx. 1.5% of body weight on a feed dry weight: lobster wet weight basis. Belt feeders were loaded once per day with a pre-weighed ration of feed derived from the initial total lobster biomass in each aquarium. At the mid-point of the experiment (4 weeks), a bulk-weighing (total wet weight of lobsters per aquarium) was performed to adjust feed rations relative to biomass changes due to growth and to mortality. Uneaten feeds were collected daily for determination of apparent feed intake (AFI), and each aquarium was cleaned by siphon before the subsequent feed period. Apparent feed intake was measured in situ as previously described^28,32^. Uneaten feed was collected on a 125 µm mesh screen, rinsed with deionised water to remove salts, weighed and stored frozen at − 20 °C in a cumulative weekly sample. Dry matter content of weekly uneaten feed composite samples (n = 3 per feed treatment per week) was subsequently determined after oven drying at 105 °C for 24 h^67^ for input into daily AFI calculations. To correct AFI for feed DM losses, a feed leachability assessment was determined under the normal experimental conditions but in the absence of experimental lobsters. For the leachability assessment, a typical daily feed ration was delivered to each aquarium by belt feeder (n = 3 per feed treatment) over 16 h as during the experiment. Consistent with the daily experimental routine and timing, feeds were then removed from each aquarium by siphon, collected on a 125 µm mesh screen, rinsed with deionised water and oven dried at 105 °C for 24 h for DM determination.

### Chemical analyses

#### Chemical composition analysis

Frozen samples were freeze-dried (FD) to a constant weight and then pooled by tissue/ sample type (experimental feeds, lobster carcass, HP and TM tissues) and treatment replicate. All FD samples were stored at − 80 °C prior to analysis. Dry matter content of FD samples was determined gravimetrically after oven drying at 105 °C for 24 h^67^. All biochemical analyses were performed on finely ground FD samples and corrected for DM. Ash content was determined by combustion of FD samples in a furnace at 600 °C for 2 h^68^. Crude protein content was determined after measuring elemental nitrogen (N) composition of FD samples using flash combustion isotope ratio mass spectrometry (Elementar vario PYRO cube coupled to an isoprime 100 mass spectrometer) at the Central Science Laboratory, University of Tasmania, Australia as described by Marchese et al.^69^. A conversion factor of 6.25 x %N was used to calculate crude protein (CP) content. Total lipid content of FD samples was determined gravimetrically using a modified Bligh and Dyer^70^ method. Total lipid (TL) was extracted in a mixture of chloroform, methanol and milli-Q water (1:1:0.9 v/v/v) as described by Yagiz et al.^71^. The total choline content of feeds was determined by Upscience Labs Solutions (https://www.upscience-labs.com/about-us/our-worldwide-presence/vietnam/) according to in-house protocols. Bomb calorimetry was used to determine the gross energy (GE) content of FD samples according to manufacturer specifications (Parr 6400 Automatic Isoperibol Calorimeter, IL, USA).

#### Lipid class analyses

An aliquot of total lipid (10–20 mg in 1 mL chloroform) was spotted on Chromarods S-V and developed in hexane:diethylether:acetic acid (70:10:0.1 v/v/v) and dried for 10 min at 80 °C. After drying, pyrolysis of Chromarods were performed by an Iatroscan™ MK-5 thin-layer chromatography-flame ionization detector analyser (Mitsubishi Kagaku Iatron, Inc., Japan). Peak areas were quantified by SIC-480II for Iatroscan™ Integrating Software v.7.0-E (System Instruments Co., Mitsubishi Chemical Medience Corporation, Japan). Lipid class was identified by comparison with corresponding standards.

#### Cholesterol determination

Quantitative Chol measurements were performed on aliquots of total lipid extracts (equivalent to approx. 3 mg total lipid per sample). A surrogate standard of 10 µg D7-Chol (Cat. 25265, Sapphire Biosciences Pty Ltd, Redfern, NSW, Australia) was added to each sample prior to saponification and extraction. Samples were evaporated to dryness at 35 °C under a stream of nitrogen gas. Total lipid extracts were saponified using 3 mL of 1% KOH in 80% methanol and heating for 3 h at 80 °C. Samples were cooled to room temperature, 1 mL milliQ water added and then mixed thoroughly. Extraction was performed in 1.5 mL of 4:1 (v/v) hexane: chloroform extraction solvent. Samples were mixed thoroughly, left to stand to allow phase separation and the upper phase then removed to a clean vial. The extraction step was repeated a further two times and the upper phase extracts combined. Combined extracts were quantitatively transferred to GC vials and evaporated to dryness at 35 °C under a stream of nitrogen gas. Fifty microlitres of chloroform together with 50 µL of BSTFA (containing 1% TMCS) was added and samples heated at 60 °C overnight. Samples were then cooled, evaporated to dryness at 35 °C under a stream of nitrogen gas and made to 100 µL with chloroform.

Cholesterol was analysed using a Varian CP-3800 gas chromatograph coupled to a Bruker 300MS triple quadrupole mass spectrometer (Bruker Corporation, Massachusetts, USA) fitted with an Agilent DB-5MS column (30 m × 0.25 mm; 0.25 µm film thickness). Helium was used as the carrier gas with a flow rate of 1.2 mL min^−1^. The Injector was set to 290 °C and the Transfer line to 290 °C. Samples were injected at 45 °C in split mode (10:1). After 1 min, the oven was programmed from 45 to 120 °C at 30 °C min^−1^, then at 4 °C min^−1^ to 320 °C, which was held for 20 min. Injection volume was 1 µL. Electron ionisation mass spectra were recorded in full scan mode over the range (m/z) 40 to 600. Single ion monitoring (SIM) acquisitions were also used as the following: Chol-TMS (m/z) 329.6, 368.6, 458.4; D7-Chol-TMS (m/z) 336.6, 375.6, 465.6. Identity of Chol was confirmed by comparison to the stable isotope labelled surrogate standard and the NIST2017 Mass Spectral Library (National Institute of Standards and Technology, USA). Data was processed using MS Workstation Version 7.

### Calculations and statistical analysis

All calculations were performed as previously described^28^. Normality and homogeneity of variance were assessed using suitable tests (e.g., Shapiro–Wilk’s and Levene’s tests). Proportional data were arcsine square root transformed prior to analysis. For other data, log_10_ transformations were used as required. Statistical comparisons were made by two-way analysis of variance (ANOVA) using feed Chol and PhosL as factors. Where statistically significant effects were observed, Tukey’s post-hoc analysis was performed to establish significant differences between means. All statistical analyses were performed using the jamovi project (2020) R GUI jamovi software (v1.6.23) (retrieved from https://www.jamovi.org). The critical level of statistical significance for all tests was α = 0.05.

### Ethics declarations

Animal ethics declaration made in the body of this manuscript under the Animals subsection of the Methods. “Lobster husbandry and experimental research was conducted within the general framework and principles of the Australian Code for the Care and Use of Animals for Scientific Purposes (2013). The University of Tasmania does not require Animal Ethics Committee approval for research conducted on crustaceans based on the Tasmanian Animal Welfare Act 1993 (https://www.legislation.tas.gov.au/view/whole/html/inforce/current/act-1993-063), which stipulates that Animal Ethics Committee approval is required for animal research conducted on living, non-human vertebrates and cephalopods. ”

## Data Availability

The datasets generated during and/ or analysed during the current study are available from the corresponding author on reasonable request.

## References

[1] Goad LJ (1981). Sterol biosynthesis and metabolism in marine invertebrates. Pure Appl. Chem..

[2] Kanazawa A (2001). Sterols in marine invertebrates. Fish. Sci..

[3] Kumar V (2018). Metabolism and nutritive role of cholesterol in the growth, gonadal development, and reproduction of crustaceans. Rev. Fish. Sci. Aquac..

[4] Mykles DL (2011). Ecdysteroid metabolism in crustaceans. J. Steroid Biochem. Mol. Biol..

[5] NRC. National Research Council. *Nutrient Requirements of Fish and Shrimp* (The National Academies Press, 2011).

[6] Zhang W (2019). Study of the requirements of dietary cholesterol at two different growth stages of Pacific white shrimps, Litopenaeus vannamei. Aquacult. Int..

[7] Briggs MRP, Jauncey K, Brown JH (1988). The cholesterol and lecithin requirements of juvenile prawn (Macrobrachium rosenbergii) fed semi-purified diets. Aquaculture.

[8] Chen H-Y (1993). Requirements of marine shrimp, Penaeus monodon, juveniles for phosphatidylcholine and cholesterol. Aquaculture.

[9] D'Abramo LR (1998). Nutritional requirements of the freshwater prawn Macrobrachium rosenbergii: Comparisons with species of Penaeid shrimp. Rev. Fish. Sci..

[10] Teshima S, Ishikawa M, Koshio S, Kanazawa A (1997). Assessment of cholesterol requirements in the prawn, Penaeus japonicus. Aquac. Nutr..

[11] Holme M-H, Zeng C, Southgate PC (2006). The effects of supplemental dietary cholesterol on growth, development and survival of mud crab, Scylla serrata, megalopa fed semi-purified diets. Aquaculture.

[12] Noordin NM, Zeng C, Southgate PC (2020). Survival, molting pattern, and growth of early blue swimmer crab, Portunus pelagicus, juveniles fed diets containing varying levels of cholesterol. J. World Aquac. Soc..

[13] Sheen S-S (2000). Dietary cholesterol requirement of juvenile mud crab Scylla serrata. Aquaculture.

[14] Suprayudi MA, Takeuchi T, Hamasaki K (2012). Cholesterol effect on survival and development of larval mud crab Scylla serrata. HAYATI J. Biosci..

[15] Tao X (2014). Effects of dietary cholesterol levels on moulting performance, lipid accumulation, ecdysteroid concentration and immune enzymes activities of juvenile Chinese mitten crab Eriocheir sinensis. Aquac. Nutr..

[16] D'Abramo LR, Wright JS, Wright KH, Bordner CE, Conklin DE (1985). Sterol requirement of cultured juvenile crayfish, Pacifastacus leniusculus. Aquaculture.

[17] Hernández PV, Olvera-Novoa MA, Rouse DB (2004). Effect of dietary cholesterol on growth and survival of juvenile redclaw crayfish Cherax quadricarinatus under laboratory conditions. Aquaculture.

[18] Bordner CE, D'Abramo LR, Conklin DE, Baum NA (1986). Development and evaluation of diets for crustacean aquaculture. J. World Aquac. Soc..

[19] Castell JD, Covey JF (1976). Dietary lipid requirements of adult lobsters, Homarus americanus (M.E.). J. Nutr..

[20] Castell JD, Mason EG, Covey JF (1975). Cholesterol requirements of juvenile American lobster (Homarus americanus). J. Fish. Res. Board Can..

[21] D'Abramo LR, Bordner CE, Conklin DE, Baum NA (1984). Sterol requirement of juvenile lobsters, Homarus sp. Aquaculture.

[22] Irvin SJ, Williams KC, Barclay MC, Tabrett SJ (2010). Do formulated feeds for juvenile Panulirus ornatus lobsters require dietary cholesterol supplementation?. Aquaculture.

[23] Gong H, Lawrence AL, Jiang D-H, Castille FL, Gatlin DM (2000). Lipid nutrition of juvenile Litopenaeus vannamei: I. Dietary cholesterol and de-oiled soy lecithin requirements and their interaction. Aquaculture.

[24] Niu J (2012). Excess dietary cholesterol may have an adverse effect on growth performance of early post-larval Litopenaeus vannamei. J. Anim. Sci. Biotechnol..

[25] Thongrod S, Boonyaratpalin M (1998). Cholesterol and lecithin requirement of juvenile banana shrimp. Penaeus merguiensis. Aquaculture.

[26] Morris TC, Samocha TM, Davis DA, Fox JM (2011). Cholesterol supplements for Litopenaeus vannamei reared on plant based diets in the presence of natural productivity. Aquaculture.

[27] Coutteau P, Geurden I, Camara MR, Bergot P, Sorgeloos P (1997). Review on the dietary effects of phospholipids in fish and crustacean larviculture. Aquaculture.

[28] Landman MJ, Codabaccus BM, Fitzgibbon QP, Smith GG, Carter CG (2021). Fresh or formulated: A preliminary evaluation of fresh blue mussel (Mytilus galloprovincialis) and formulated experimental feeds with inclusion of fresh blue mussel on the growth performance of hatchery-reared juvenile slipper lobster (Thenus australiensis). Aquaculture.

[29] Jeffs A, Hooker S (2000). Economic feasibility of aquaculture of spiny lobsters Jasus edwardsii in temperate waters. J. World Aquac. Soc..

[30] Landman MJ (2020). Physiological status and nutritional condition of cultured juvenile Thenus australiensis over the moult cycle. Comp. Biochem. Physiol. B.

[31] Vijayakumaran M, Radhakrishnan EV (2011). Recent Advances and New Species in Aquaculture.

[32] Landman MJ, Codabaccus BM, Carter CG, Fitzgibbon QP, Smith GG (2021). Is dietary phosphatidylcholine essential for juvenile slipper lobster (Thenus australiensis)?. Aquaculture.

[33] Wirtz A (2022). Protein sources influence both apparent digestibility and gastrointestinal evacuation rate in juvenile slipper lobster (Thenus australiensis). Comp. Biochem. Physiol. A.

[34] Chang ES, Mykles DL (2011). Regulation of crustacean molting: A review and our perspectives. Gen. Comp. Endocrinol..

[35] Nagaraju GP (2011). Reproductive regulators in decapod crustaceans: An overview. J. Exp. Biol..

[36] Subramoniam T (2000). Crustacean ecdysteriods in reproduction and embryogenesis. Comp. Biochem. Physiol. Part C.

[37] D'Abramo LR, Bordner CE, Conklin DE (1982). Relationship between dietary phosphatidylcholine and serum cholesterol in the lobster Homarus sp. Mar. Biol..

[38] Deshimaru O, Kuroki K (1974). Studies on a purified diet for prawn-II: Optimum contents of cholesterol and glucosamine in the diet. Nippon Suisan Gakkaishi.

[39] Kanazawa A, Tanaka N, Teshima S, Kashiwada K (1971). Nutritional requirements of prawn. II. Requirement for sterols. Nippon Suisan Gakkaishi.

[40] Shudo K, Nakamura K, Ishikawa S, Kitabayashi K (1971). Studies on formula feed for Kuruma prawn. IV. On the growth promoting effect of both squid liver oil and cholesterol. Bull. Tokai Reg. Fish. Res. Lab..

[41] Teshima S-I, Kanazawa A, Sasada H, Kawasaki M (1982). Requirements of the larval prawn, Penaeus japonicus, for cholesterol and soybean phospholipids. Mem. Fac. Fish. Kagoshima Univ..

[42] Teshima S-I, Ishikawa M, Koshio S, Kanazawa A (1997). Necessity of dietary cholesterol for the freshwater prawn. Fish. Sci..

[43] Sheen S-S, Liu P-C, Chen S-N, Chen J-C (1994). Cholesterol requirement of juvenile tiger shrimp (Penaeus monodon). Aquaculture.

[44] Smith DM, Tabrett SJ, Barclay MC (2001). Cholesterol requirement of subadult black tiger shrimp Penaeus monodon (Fabricius). Aquac. Res..

[45] Johnston DJ, Yellowlees D (1998). Relationship between dietary preferences and digestive enzyme complement of the slipper lobster Thenus orientalis (Decapoda: Scyllaridae). J. Crustacean Biol..

[46] Spanier, E. & Lavalli, K. L. *Lobsters: Biology, Management, Aquaculture and Fisheries* 414–466 (2013).

[47] Gong H, Lawrence AL, Jiang D-H, Gatlin DM (2000). Lipid nutrition of juvenile Litopenaeus vannamei II. Active components of soybean lecithin. Aquaculture.

[48] Francis DS, Salmon ML, Kenway MJ, Hall MR (2014). Palinurid lobster aquaculture: Nutritional progress and considerations for successful larval rearing. Rev. Aquac..

[49] Smith GG, Ritar AJ, Johnston D, Dunstan GA (2004). Influence of diet on broodstock lipid and fatty acid composition and larval competency in the spiny lobster, Jasus edwardsii. Aquaculture.

[50] Chang ES (1995). Physiological and biochemical changes during the molt cycle in decapod crustaceans: An overview. J. Exp. Mar. Biol. Ecol..

[51] Chang ES, Chang SA, Mulder EP (2001). Hormones in the lives of crustaceans: An overview. Am. Zool..

[52] McLeod LE, Carter CG, Johnston DJ (2004). Changes in the body composition of the adult male southern rock lobster, Jasus edwardsii, during starvation. J. Shellfish Res..

[53] Vinagre AS, Chung JS (2016). Effects of starvation on energy metabolism and crustacean hyperglycemic hormone (CHH) of the Atlantic ghost crab Ocypode quadrata (Fabricius, 1787). Mar. Biol..

[54] Berry SE, Simon CJ, Foote AR, Jerry DR, Wade NM (2019). Evaluation of baseline haemolymph biochemistry, volume and total body energetics to determine an accurate condition index in the black tiger shrimp, Penaeus monodon. Comp. Biochem. Physiol. B. Biochem. Mol. Biol..

[55] Coutteau P, Kontara EKM, Sorgeloos P (2000). Comparison of phosphatidylcholine purified from soybean and marine fish roe in the diet of postlarval Penaeus vannamei Boone. Aquaculture.

[56] Baum NA, Conklin DE, Chang ES (1990). Effect of dietary lecithin in combination with casein or crab protein on cholesterol uptake and transport in the lobster Homarus americanus. J. World Aquac. Soc..

[57] D'Abramo LR, Baum NA, Bordner CE, Conklin DE, Chang ES (1985). Diet-dependent cholesterol transport in the American lobster. J. Exp. Mar. Biol. Ecol..

[58] Li X-Y (2016). Effects of phospholipid addition to diets with different inclusion levels of fish oil on growth and fatty acid body composition of juvenile swimming crab Portunus trituberculatus. Aquac. Res..

[59] Teshima S-I, Kanazawa A (1980). Transport of dietary lipids and role of serum lipoproteins in the prawn. Nippon Suisan Gakkaishi.

[60] Teshima S-I, Kanazawa A (1980). Lipid constituents of serum lipoproteins in the prawn. Nippon Suisan Gakkaishi.

[61] Teshima S-I, Kanazawa A, Kakuta Y (1986). Effects of dietary phospholipids on growth and body composition of the juvenile prawn. Nippon Suisan Gakkaishi.

[62] Teshima S-I, Kanazawa A, Kakuta Y (1986). Role of dietary phospholipids in the transport of (14C) tripalmitin in the prawn. Nippon Suisan Gakkaishi.

[63] NHMRC (2013). Australian Code for the Care and Use of Animals for Scientific Purposes.

[64] Fitzgibbon QP, Battaglene SC (2012). Effect of photoperiod on the culture of early-stage phyllosoma and metamorphosis of spiny lobster (Sagmariasus verreauxi). Aquaculture.

[65] Jensen MA, Carter CG, Adams LR, Fitzgibbon QP (2013). Growth and biochemistry of the spiny lobster Sagmariasus verreauxi cultured at low and high density from hatch to puerulus. Aquaculture.

[66] Shu-Chien AC (2017). Effect of dietary lipid source on expression of lipid metabolism genes and tissue lipid profile in juvenile spiny lobster Sagmariasus verreauxi. Aquaculture.

[67] AOAC (1999). Official Methods of Analysis of AOAC International.

[68] AOAC (1995). Official Methods of Analysis of AOAC International.

[69] Marchese G (2019). The influence of flesh ingredients format and krill meal on growth and feeding behaviour of juvenile tropical spiny lobster Panulirus ornatus. Aquaculture.

[70] Bligh EG, Dyer WJ (1959). A rapid method of total lipid extraction and purification. Can. J. Biochem. Physiol..

[71] Yagiz Y (2009). Effect of high-pressure processing and cooking treatment on the quality of Atlantic salmon. Food Chem..

